# The Application of Peptide Nucleic Acids (PNA) in the Inhibition of Proprotein Convertase Subtilisin/Kexin 9 (*PCSK9*) Gene Expression in a Cell-Free Transcription/Translation System

**DOI:** 10.3390/ijms25031463

**Published:** 2024-01-25

**Authors:** Agnieszka Polak, Grzegorz Machnik, Łukasz Bułdak, Jarosław Ruczyński, Katarzyna Prochera, Oliwia Bujak, Piotr Mucha, Piotr Rekowski, Bogusław Okopień

**Affiliations:** 1 Department of Internal Medicine and Clinical Pharmacology, Faculty of Medical Science in Katowice, Medical University of Silesia, Medykow 18, 40-752 Katowice, Poland; 2 Laboratory of Chemistry of Biologically Active Compounds, Faculty of Chemistry, University of Gdańsk, Wita Stwosza 63, 80-308 Gdańsk, Poland; jaroslaw.ruczynski@ug.edu.pl (J.R.); katarzyna.prochera@phdstud.ug.edu.pl (K.P.);

**Keywords:** proprotein convertase subtilisin/kexin 9 (PCSK9), peptide nucleic acids (PNA), antigene activity, transcription, translation

## Abstract

Proprotein convertase subtilisin/kexin 9 (PCSK9) is a protein that plays a key role in the metabolism of low-density lipoprotein (LDL) cholesterol. The gain-of-function mutations of the *PCSK9* gene lead to a reduced number of surface LDL receptors by binding to them, eventually leading to endosomal degradation. This, in turn, is the culprit of hypercholesterolemia, resulting in accelerated atherogenesis. The modern treatment for hypercholesterolemia encompasses the use of biological drugs against PCSK9, like monoclonal antibodies and gene expression modulators such as inclisiran—a short, interfering RNA (siRNA). Peptide nucleic acid (PNA) is a synthetic analog of nucleic acid that possesses a synthetic peptide skeleton instead of a phosphate–sugar one. This different structure determines the unique properties of PNA (e.g., neutral charge, enzymatic resistance, and an enormously high affinity with complementary DNA and RNA). Therefore, it might be possible to use PNA against PCSK9 in the treatment of hypercholesterolemia. We sought to explore the impact of three selected PNA oligomers on *PCSK9* gene expression. Using a cell-free transcription/translation system, we showed that one of the tested PNA strands was able to reduce the *PCSK9* gene expression down to 74%, 64%, and 68%, as measured by RT–real-time PCR, Western blot, and HPLC, respectively. This preliminary study shows the high applicability of a cell-free enzymatic environment as an efficient tool in the initial evaluation of biologically active PNA molecules in the field of hypercholesterolemia research. This cell-free approach allows for the omission of the hurdles associated with transmembrane PNA transportation at the early stage of PNA selection.

## 1. Introduction

Atherosclerosis is the main cause of deaths and comorbidities worldwide. Annually, as many as 17.5 million people die due to cardiovascular diseases. Atherosclerosis is a chronic inflammatory disease of the arteries, characterized by the formation of atherosclerotic plaques in their walls. This process begins at an early stage of life and lasts its entirety. It may be asymptomatic or can be responsible for typical symptoms of cardiovascular disease, such as angina pectoris, intermittent claudication, and neurological deficiencies [1]. Atherosclerotic plaques are characterized by the progressive accumulation of cholesterol and infiltration of monocytes into the arterial wall. Monocytes, after their transformation into macrophages, absorb low-density lipoproteins (LDL) in the vascular wall, thus becoming foam cells [2]. Subsequently, a narrowing of the arterial lumen is seen, causing ischemia and eventually resulting in cardiovascular incidents due to plaque instability [3]. Among cardiovascular risk factors, LDL cholesterol (LDL-C) plays a key role in the triggering and progression of atherosclerosis. The concentration of LDL cholesterol in the blood largely depends on the number of LDL cholesterol receptors (LDLR) present on the surface of hepatocytes (responsible for the uptake of LDL-C from the blood stream into the liver) and on the activity of the proprotein convertase subtilisin/kexin type 9 enzyme (PCSK9), which affects the amount of LDL-R on liver cells [4,5,6].

PCSK9 belongs to the family of subtilisin proteases [7]. PCSK9 consists of a signal peptide, N-terminal prodomain, catalytic domain, and C-terminal domain [8] forming a protein with a mass of 72 kDa and 692 amino acid residues. The *PCSK9* gene is located on chromosome 1 (1q32.3) and contains 13 exons [9]. Gene and protein sequences are deposited in (https://www.ncbi.nlm.nih.gov/gene/255738) and (https://www.uniprot.org/uniprotkb/Q8NBP7), accessed on 15 November 2023, respectively.

**Figure 1:**
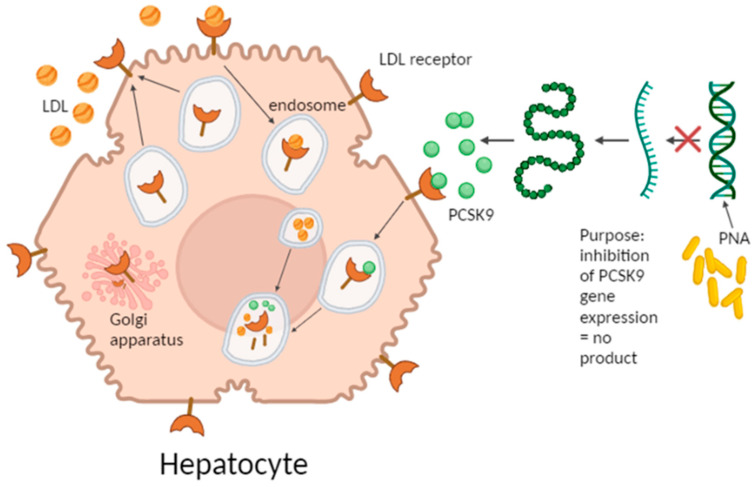
Schematic graph presenting the crucial role of proprotein convertase subtilisin/kexin type 9 (PCSK9) on LDL receptor turnover in hepatocytes (created with BioRender.com, accessed on 15 November 2023).

The role of the *PCSK9* gene in lipid metabolism was discovered in 2003 [10,11,12]. It was found that people who had a *PCSK9* gene mutation resulting in its loss of function (LOF) exhibited a reduced risk of coronary disease due to the low blood LDL-C level [13]. Hepatic PCSK9 is a protein that regulates the half-life of LDLR in the liver. Typically, PCSK9 attaches to LDLR, and this process destines a simultaneous degradation of LDLR inside endosomes (Figure 1). This results in a reduction in the number of LDLR receptors on the hepatocyte surface, reducing uptake of LDL-C by the liver and, thus, the increase in the blood LDL cholesterol level [4,14,15]. When PCSK9 is not present, LDLRs are abundantly available on hepatocytes’ surfaces and bind LDL on the surfaces of hepatocytes. This leads to the endosomal digestion of LDLs, but LDLR are able return to the cell surface and bind further LDL molecules, leading to a reduced level of LDL-C in the serum.

Peptide nucleic acids (PNA) are molecules with a wide range of applications. PNA is a synthetic analogue of nucleic acid in which the sugar phosphate backbone has been replaced by a synthetic peptide backbone, most often consisting of N-(2-aminoethyl)-glycine units connected by amide bonds [16]. Unlike DNA and its other analogues, PNA does not contain any sugar residues or phosphate groups. This different structure determines the unique properties of PNA, which include acyclicity, achirality, and electrical neutrality [17]. Although PNA is similar to DNA molecules in many ways, in some aspects, it shows advantageous properties [18]. These include greater chemical stability and a high resistance to hydrolytic enzymes, resulting in slower degradation [19]. Importantly, PNA can also specifically bind to DNA and RNA, forming exceptionally heat-resistant and stable complexes [20]. In this context, PNA is considered a compound exerting potential antisense or antigenic action. Thanks to its properties, it may constitute a new therapeutic approach for the treatment of cardiovascular diseases.

PCSK9 is a promising target in lipid-lowering therapies. LDL-C is a major risk factor in cardiovascular diseases. It has been observed that some mutations found within the *PCSK9* gene are associated with hypercholesterolemia and with an increased risk of cardiovascular diseases. These missense, gain-of-function (GOF) genetic variants cause a constitutive activation of the *PCSK9* gene, thus providing LDL-C levels in the blood pf over 190 mg/dL in adults. Although numerous PCSK9 GOF variants have been found and their frequencies differ between populations, the most known variants are as follows: E32K, L108R, S127R, D129G, D129N, R218S, and D374Y (protein reference sequence: Q8NBP7-1) [21]. Clinical studies have shown that inhibition of PCSK9 in combination with statins significantly lowers LDL-C levels in patients’ blood [22,23].

Several research groups have examined numerous PCSK9-inhibitory molecules with therapeutic potential. These encompass monoclonal antibodies (alirocumab, evolokumab, bococizumab) and the small interfering RNA (siRNA) inlisiran [24].

Peptide nucleic acid oligomers may constitute a promising approach to this topic, mainly because of their chemical advantages of having a strong resistance to hydrolysis and a neutral charge. Therefore, we conceived an experiment to explore the potential application of PNA-based molecules on the inhibition of the expression of PCSK9. In our study, three different PNA oligomers targeting distant sequences of the *PCSK9* gene were investigated. We used a cell-free in vitro translation system for the determination of the most effective DNA target within the *PCSK9* gene and optimization of the PNA-to-DNA ratio. The results of these studies might provide a basis for consecutive tests in human cell culture systems.

## 2. Results

### 2.1. Capillary Electrophoresis Analysis of Interaction of PNA-Ahx-NLS with Its Complementary DNA Fragments

**Figure 2:**
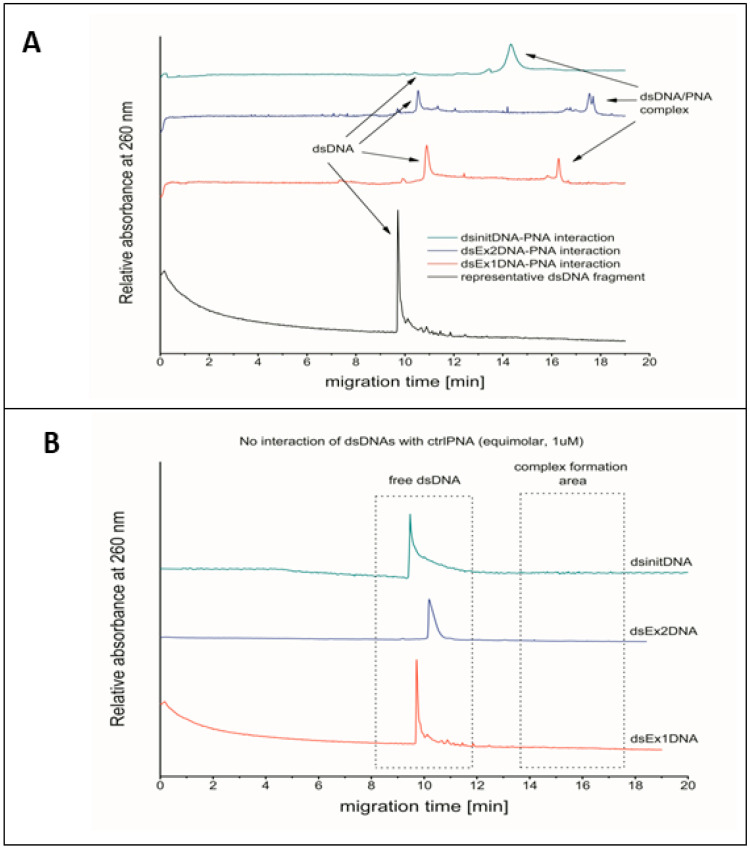
(**A**) Capillary electrophoresis analysis of interaction of PNA with its complementary DNA fragments. UV absorbance was measured at a wavelength of 260 nm. Absorbance intensities of DNA interactions with different complementary PNA fragments are depicted as green, blue, and red lines for InitPNA, Ex2PNA, and Ex1PNA, respectively. (**B**) Electropherograms of dsDNA fragments without addition of PNA.

There were two distant peaks observed in capillary electrophoresis of PNA and its complementary DNA fragments: one was specific to PNA/DNA hybrids and another one to double-stranded DNA (dsDNA) (Figure 2). This was true for all kinds of PNA oligomers, i.e., for InitPNA, Ex1PNA, and Ex2PNA. In a control sample without the addition of PNA, only a peak corresponding to double-stranded DNA fragments was detected. No specific interaction between PNA and DNA was found. These results confirm the predicted complementarity and affinity effects of PNA molecules to the target sequence of the *PCSK9* gene.

### 2.2. Reverse Transcription Real-Time Polymerase Chain Reaction (RT–Real-Time PCR) and Conventional PCR

**Figure 3:**
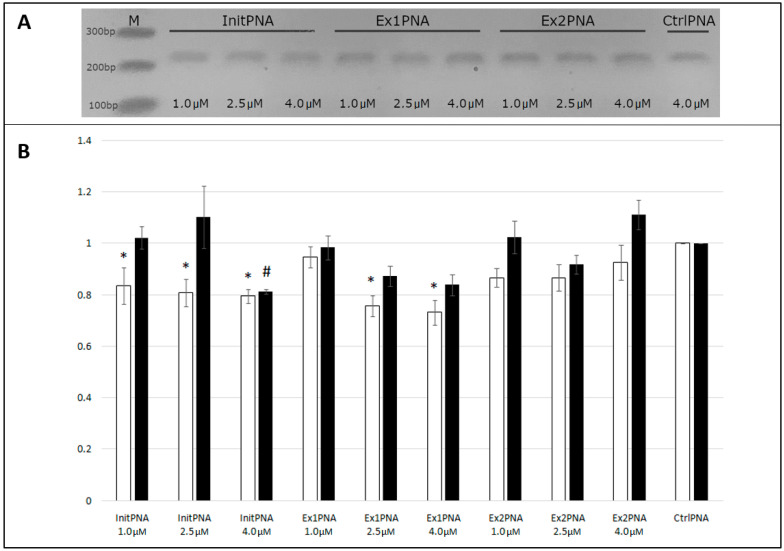
Integrated optical density (IOD) of DNA bands after conventional reverse transcription PCR (RT–PCR) (**B**) and agarose gel electrophoresis (**A**). An equal amount of RNA taken from the T7 RNA polymerase reaction served as a template. The control sample contained a PNA oligomer that was not complementary to the PCSK9 coding sequence. This sample was arbitrarily assigned a value of 100%. In Panel (**B**), dashed line bars represent real-time PCR, while white dotted black bars represent conventional PCR after 15 cycles, in comparison to the control PNA. *—*p* < 0.05 vs. ctrlPNA for RT-PCR, #—*p* < 0.05 vs. ctrlPNA for conventional PCR.

According to the results obtained via real-time PCR, we found that PNA oligomers influenced the reaction efficiency of T7 RNA polymerase, albeit to a different extent. The results are expressed as the relative RNA amount measured in a sample in comparison to a sample that contained a non-complementary PNA to *PCSK9* gene according to the formula 2^−ΔCt^, where ΔCt = Ct_template+PNA_ − Ct_template_. The Ct value of a sample indicates the reaction cycle number where the fluorescent signal exceeds the threshold level. Our analysis revealed that PNA oligomers targeted to the exon 2 of *PCSK9* gene (Ex2PNA) did not show any significant inhibitory potential in comparison to the expression level observed in the control sample. Conversely, those PNA oligomers that targeted the transcription initiation codon of the *PCSK9* gene (InitPNA) and those targeted within exon1 showed an anti-PCSK9 suppression effect, and the strength of inhibition was concentration-dependent (Figure 3). The expression levels were lowered to 80% and 74% (both *p* < 0.05 vs. CtrlPNA) of that for the control sample for InitPNA and Ex1PNA, respectively.

A similar tendency was observed in the case of conventional PCR analysis. This assay was performed in order to visually confirm the results obtained using the real-time PCR technique. The polymerase chain reaction consisted of 15 cycles in order to observe the results from the logarithmic phase of product amplification. Again, PNA oligomers that targeted the transcription initiation codon of *PCSK9* gene (InitPNA), as well as those complementary to the sequence of exon1 (Ex1PNA), showed lower product load than that in the control sample (CtrlPNA), thus indicating the suppression of PCSK9 transcription (Figure 3). An amount of amplification products was measured in an agarose gel picture and was calculated as the area under the intensity peak. We observed that the strength of inhibition depended on the PNA concentration, as shown in the real-time PCR assay. Additionally, the conventional PCR analysis confirmed the specificity of the reaction, as only one clear band was observed in the gel, without any unspecific by-products.

### 2.3. Western Blot

**Figure 4:**
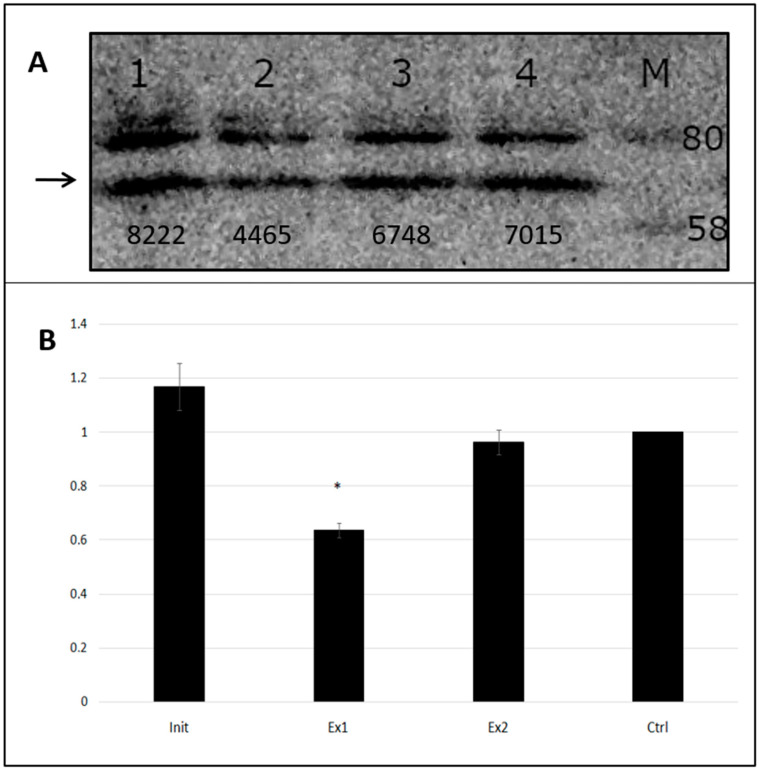
Western blot image of an in vitro transcription/translation reaction product resolved by electrophoresis with appropriate integrated optical density (I.O.D) values (**A**) and the relative amount of protein with respect to control sample (**B**). 1–4: Samples that contained InitPNA, Ex1PNA, Ex2PNA, and control (non-PCSK9 specific) PNA, respectively. M: Color Prestained Protein Standard, Broad Range (New England Biolabs, no. Cat. P7712). The PCSK9-specific band is depicted with an arrow. A total amount of 10 µg (25 µL sample volume) of purified products from the transcription/translation reaction was loaded onto the gel. The initial protein concentration in the samples was assessed spectrophotometrically. Measurements were made using ImageJ 1.53t software [25,26] *: *p* < 0.05 vs. control.

The Western blot technique allowed for the use of either PCSK9- or GFP-specific antibodies to estimate the relative protein expression level in a cell-free transcription/translation system. This was possible because an intact pCMV6-AC-GFP plasmid was utilized as a template, so the newly synthesized PCSK9 protein possessed a GFP tag at its C-end. We observed that the addition of peptide nucleic acid oligomers to the transcription/translation enzymatic machinery resulted in differences in protein expression. The presence of specific PNA altered the reaction yield depending on the PNA type. According to the integrated optical value (IOD), the lowest protein level was observed in the sample containing Ex1PNA, while InitPNA and Ex2PNA either did not reduce the PCSK9 protein level at all or affected it to a minimal extent (Figure 4). Similar results were obtained regardless of the antibody used, i.e., both anti-PCSK9 and anti-GFP antibodies. A maximum inhibition level of PCSK9 expression was observed in the Ex1PNA-containing sample to reach 64% (*p* < 0.05 vs. CtrlPNA) of the expression in a control sample. A total of 25 µL of purified products from the transcription/translation reaction was loaded onto polyacrylamide gel.

### 2.4. High-Performance Liquid Chromatography (HPLC)

**Figure 5:**
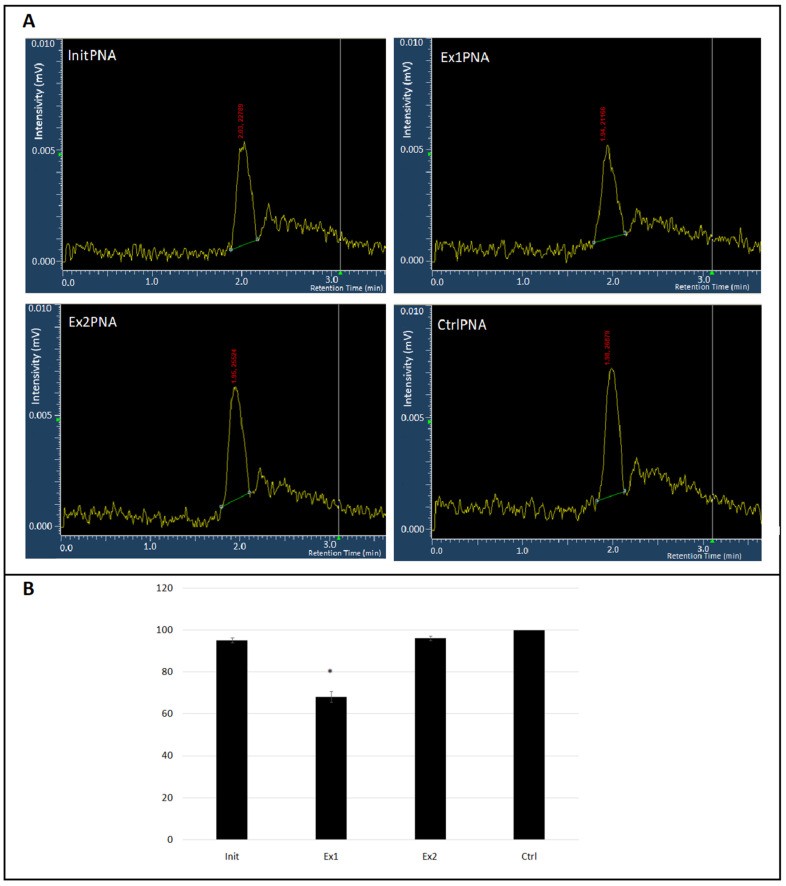
The representative HPLC traces of GFP-tagged PCSK9 synthetic protein after addition of PNA oligomers (**A**). Area under the curve (AUC) values were calculated for each sample (run in triplicates) from chromatography peaks of GFP-tagged PCSK9 protein synthesized with the presence of different PNA oligomers (**B**) *: *p* < 0.05 vs. CtrlPNA.

The samples that were taken for Western blot analysis were also subsequently used for HPLC. This assay was performed using a Merck/Hitachi D-7000 HPLC system along with a Hitachi/Merck LaChrom L-7485 Fluorescence Detector. A fluorescence-based approach was possible due to the presence of a GFP (green fluorescent protein) tag adjacent to the PCSK9 synthetic protein. Therefore, only clear and single peaks were observed in chromatograms that were not disturbed by any by-products or by reaction mixture components. The retention time (RT value) for GFP-tagged PCSK9 was 3.05 min. An area under the peak was calculated for all samples using D-7000 HSM instrument software version 4.1 in order to calculate the total protein quantity in a sample. Each reaction mix was analyzed in triplicate (three injections of 15 µL each were made). The results of our HPLC analysis unambiguously confirmed those of Western blotting, documenting that the most effective inhibitory potential against *PCSK9* gene expression was presented by the Ex1PNA molecule (Figure 5).

As shown by the amplification techniques and by Western blot analysis, the relative amount of PCSK9 protein was diminished in the sample containing Ex1PNA (down to 68% with respect to the control sample; *p* < 0.05).

## 3. Discussion

Among different gene-silencing approaches, peptide nucleic acid (PNA) technology is still seriously taken into consideration. Although more than 30 years have passed since its discovery by Professor Nielsen and his group, PNA oligomers can be mostly found in anti-bacterial investigations, as a part of molecular detection assays (PNA probes), or for the selective clamping of unwanted templates from complex mixtures [27,28,29]. Nevertheless, the strong potential antigene action displayed by PNA may supplement the repertoire of gene-targeted therapeutics with inhibiting potential.

We analyzed three distinct PNA oligomers, but only one of them displayed reasonably positive features to be considered for further analysis. Our assays revealed that the expression inhibition from PCSK9-coding plasmid DNA by Ex1PNA oligomers took place at the level of transcription, not translation. We observed a similar reduction in both RNA and protein abundance after adding Ex1PNA (down to 74%, 64%, and 68% in comparison to the control samples according to RT–real-time PCR, Western blot, and HPLC, respectively (Figure 3, Figure 4 and Figure 5). These results show similarities to those achieved by other authors, although their experiments were based on the KB-8-5 cancer line and, thus, could not be simply compared. Macadangdang et al. observed an expression reduction down to 65% vs. control cells [30].

The PNA oligomers used in the assay were designed according to the recommendations published previously. There are some universal rules for estimating the effective targets regardless of the gene of interest. Ricciardi et al. showed that a polypurine tract, if present in the targeted sequence, can bind PNA effectively by forming stable PNA/DNA/PNA triplexes [31]. Other approaches suggest, by logical implication, that the exact targeting of a transcription initiation site may successfully hinder the initiation of transcription. Authors have documented that not only PNA, but also other molecules of antigenic potential, like RNAs, efficiently target the transcription initiation codon [32].

Except for an optimal target choice, the final efficacy of nucleic-acid-based inhibitors also relies on oligonucleotide length and its composition. As we have shown in our previous paper regarding the use of PNA in the clamping of polymerase chain reactions from some (unwanted) targets, even a one-nucleotide shift of PNA targeting drastically diminishes its efficacy [29].

Peptide nucleic acid molecules differ significantly from other biologically active agents by exhibiting unique advantageous properties, such as enzymatic resistance and neutral charge. On the other hand, PNA shows a poor ability to pass through biological membranes. Until now, several solutions to overcome these hurdles have been developed; some of them encompass modifications of the PNA oligomers themselves, e.g., by the addition of a cell penetrating peptide (CPP) or a nucleus localization sequence (NLS), in order to improve the ability to pass through the cellular or nuclear membrane, respectively. 

Therefore, it is still reasonable to adapt and utilize a preliminary selection tool that facilitates the search for the most appropriate PNA without using a time-consuming in vitro experiment. To omit the hurdles regarding the involvement of cell lines, we effectively utilized a cell-free system that allowed for the performance of the transcription/translation steps from any plasmid-cloned DNA sequences of interest. Indeed, we tested two similar cell-free gene expression solutions, namely, “TnT Quick Coupled Transcription/Translation System” and “*E. coli* T7 S30 Extract System for Circular DNA”, both from Promega Inc., Wisconsin, MA, USA. Nevertheless, we found that the second kit was a better choice for our needs due to two reasons: 1. It allows for the synthesis of a high amount of protein that is able to be detected even using the HPLC technique; and: 2. It enables the precise estimation of the template amount that is added for reaction. The number of plasmid molecules can be easily calculated according to its molecular mass and concentration. From our point of view, a significant drawback of those cell-free systems is that they utilize T7 phage promoters for transcription, making it impossible to test any PNA targeted in the 5’ direction with respect to the transcription start codon. We designed a PNA directed against the *PCSK9* gene promoter, but it could not be tested in this experiment.

We observed that capillary electrophoresis (CE) analysis of PNA oligomers with their synthetic DNA counterparts allowed for the prediction of the potential utility of a particular PNA in biological systems. In our research, CE results indicated that PNA showed clearly detectable UV absorption that corresponded to double-stranded DNA (dsDNA) and to PNA/DNA hybrids, revealing that a specific targeted interaction occurred (Figure 5). 

The PCSK9 enzyme is known as an important factor in humans’ pathology, as this enzyme is responsible for the reduction in low-density lipoprotein receptor (LDLR) numbers on the surfaces of hepatocytes, thus eventually increasing the cholesterol level in the blood [31]. Because of its significant medical importance, numerous solutions have been investigated to achieve a reduction in the PCSK9 molecule number or activity in order to lower the hypercholesterolemia level. This is especially urgent in patients suffering from familial hypercholesterolemia where gain-of-function (GOF) mutations of the *PCSK9* gene have been observed. Indeed, a few proprotein convertase subtilisin/kexin type 9 modulators have been implemented in the therapy for hypercholesterolemia.

In this context, the unique antigene activity of peptide nucleic acid oligomers may extend the repertoire of anti-PCSK9 agents (antigene activity means that an agent influences a DNA strand or DNA strands, thus preventing transcription occurrence). For our research, we chose three different PNA oligomers that targeted distant DNA sites of a *PCSK9* gene. Our study, albeit based only on a small number of PNA oligomers, revealed that a cell-free transcription/translation system is well suited for the preliminary selection of the most effective PNA molecules that may be destined for cell-based approaches. Due to its simplicity and relatively high throughput, the capillary electrophoresis approach may additionally be helpful in PNA pre-selection, especially if a large number of oligomers have to be investigated.

## 4. Materials and Methods

### 4.1. Synthesis of PNA-Ahx-NLS Conjugates

All reagents and solvents were of analytical, HPLC, or LC-MS grade. Solutions were freshly prepared with distilled deionized water using a Milli-Q Millipore system (Bedford, MA, USA) and filtered with a 0.22 μm filter before use. Fmoc-XAL-PEG-PS resin for the PNA synthesis was obtained from Merck KGaA (Darmstadt, Germany). Fmoc/Bhoc-protected PNA monomers were purchased from Panagene (Billingham, Cleveland, UK). Fmoc-protected amino acids used for peptide synthesis were obtained from Bachem AG (Bubendorf, Switzerland). The other reagents and solvents were obtained from Sigma-Aldrich Co (Poznań, Poland).

Sequences of PNA oligomers were selected using overall recommendations concerning oligonucleotide structure, like the most effective length, purine-to-pyrimidine ratio, self-complementarity risk, etc., given by another authors. In order to achieve a strong bonding force and to establish preferable conditions for triplex-forming oligonucleotides, the presence of polypurine stretch in the target DNA was assured. In general, the most important features of the PNA design were: 1. the importance of the target on the gene functionality (e.g., the presence of transcription factor as HNF1, transcription initiation site, splicing donor/acceptor site, etc.) and 2. the presence of a polypurine tract within the DNA that facilitated the double-strand invasion by PNA (Table 1, Table 2 and Table 3) [33].

The conjugates of PNA with Ahx-NLS (Table 2) were synthesized in an automated peptide synthesizer (Quartet, Protein Technologies, Tucson, AZ, USA) by applying Fmoc chemistry [27,28]. Fmoc-XAL-PEG-PS resin (loading 0.18 mM/g) for the PNA synthesis was used as the starting material. Fmoc-protected amino acids were assembled as active derivatives in a 2.5-fold molar excess of *O*-(benzotriazole-1-yl)-1,1,3,3-tetramethyluronium tetrafluoroborate (TBTU) with the addition of *N*-hydroxybenzotriazole (HOBt) and 4-methylmorpholine (NMM) (1:1:2) in the *N,N*-dimethylformamide (DMF) solution for 2 × 30 min. In the case of coupling of the Arg residue, the derivative Fmoc-Arg(Boc)_2_-OH was used instead of the standard Fmoc-Arg(Pbf)-OH. Removal of the Fmoc group was carried out with 20% piperidine/DMF in 2 cycles (2 × 3.5 min).

After the synthesis of the Ahx-NLS sequence was completed, the Fmoc/Bhoc-protected monomers were attached as active derivatives in a 2.2-fold molar excess. PNA monomers were activated with the use of a mixture of 2-(1*H*-7-azabenzo-triazole-1-yl)-1,1,3,3-tetramethylouronium hexafluorophosphate (HATU)/NMM/2,6-lutidine (0.7:1:1.5) in DMF solution for 2 × 30 min. Removal of the Fmoc group was conducted with 20% piperidine/DMF in 2 cycles (2 × 1 min). Cleavage and deprotection of the Bhoc/Boc groups of the immobilized PNA-Ahx-NLS conjugates were performed through treatment with a mixture of trifluoroacetic acid (TFA)/*m*-cresol (70:5) in dichloromethane (DCM) solution at room temperature for 55 min under argon bubbles. The obtained crude conjugates were lyophilized, purified, and analyzed using reversed-phase high-performance liquid chromatography (HPLC).

The crude products were purified using a preparative HPLC system (SpotPrep II, Armen, Brittany, France) with a Reprosil 100 C-18 column (Dr. Maisch GmbH, Ammberbuch, Germany, 40 × 250 mm, 10 µm particle size). Several gradients of acetonitrile (ACN) with 0.08% TFA, at a flow rate of 25 mL/min, were used for purification. The column was maintained at an ambient temperature. The eluates were monitored with a UV detector at λ = 264 nm. Fractions of the highest purity (>95%) were analyzed using an analytical ultra-high-performance liquid chromatography (UHPLC)-MS system (Nexera X2 with LCMS-2020 detector, Shimadzu, Tokyo, Japan) and an Aeris WIDEPORE C4 column (Phenomenex, 100 × 2.1 mm, 3.6 µm particle size) with several gradients of can, along with the addition of 0.1% formic acid (FA) and 0.05% TFA. The column was maintained at 40 °C. The flow rate was 0.3 mL/min, and the eluates were monitored using a UV detector at λ = 264 nm and a mass spectrometry detector (ESI-MS). The identities of the compounds were confirmed via mass spectrometry (ESI-MS, Shimadzu LCMS-2020, Shimadzu Corp., Kyoto, Japan).

A nucleus localization sequence (NLS) was added to the *C*-terminus of each PNA oligomer. This was necessary due to the further PNA utilization in subsequent research steps, as PNA action strictly relies on its successful introduction into the cell nucleus (Table 2).

### 4.2. PCSK9 Plasmids

A pCMV6-AC-GFP plasmid coding for the *PCSK9* gene was purchased from OriGene Technologies (Rockville, MD, USA, Cat No. RG220000). After in vitro synthesis driven by a CMV promoter, the PCSK9 protein harbored a GFP (green fluorescent protein) at its C end, resulting in a GFP-tagged PCSK9 protein. This made the use of applications directed to GFP protein detection possible in a sample, e.g., by fluorescence detection and/or by induction of anti-GFP antibodies.

### 4.3. CAPILLARY Electrophoresis Analysis of Interaction of PNA-Ahx-NLS with Its Complementary DNA Fragments

In order to confirm the PNA complementarity and its high affinity to the target sequence of *PCSK9* gene, the specificity of PNA-Ahx-NLS hybridization to complementary DNA fragments was analyzed via capillary electrophoresis (CE) [27,28]. 

Synthetic DNA fragments of *PCSK9* gene were designed, then ordered and synthesized in Genomed SA (Warsaw, Poland).

Separations were performed using a P/ACE MDQ Plus system (SCIEX, Framingham, MA, USA) controlled by Karat software version 8.0. An uncoated fused-silica capillary (Polymicro Technologies, Phoenix, AZ, USA) of 60 (50 to detector) cm × 75 µm, thermostated at 25 °C, was used. Analyses were performed at −20 kV using a 1xTBE (pH 7.2) containing 0.5% linear polyacrylamide (LPA, av. MW 150,000) background electrolyte (BGE) with reverse electrode polarization (anode at the detector end). Samples were kept at 25 °C before analysis and introduced to the capillary at its cathodic end through electrokinetic injection at −10 kV for 10 s. Between runs, the capillary was rinsed with a new portion of BGE for 3 min. PNA/DNA hybrids were monitored with a UV detector at λ = 260 nm. All experiments were performed in triplicate.

### 4.4. In Vitro Transcription Reaction Using T7 RNA Polymerase

An in vitro transcription reaction was carried out using a T7 RNA polymerase enzyme (New England BioLabs, Ipswich, MA, USA, Cat No. M0251S). In order to prepare samples for the reaction, the pCMV6-AC-PCSK9-GFP plasmid was linearized by means of XhoI endonuclease digestion (New England BioLabs, Ipswich, MA, USA, Cat No. R0146S). An amount of 1 µg of linearized plasmid was used as a template for transcription. PCSK9-specific PNA oligomers at concentrations of 1.0, 2.5, and 4.0 µM were used for the study. These concentrations were selected according to our previous optimizations and to the literature data [29].

### 4.5. Reverse Transcription, Real-Time Polymerase Chain Reaction (RT–Real-Time PCR), and Conventional PCR

The relative amount of PCSK9 RNA obtained after T7 RNA polymerase synthesis was assessed using 1-step RT–qPCR. The reaction mix consisted of a 1× SuperScript III One-Step RT–PCR System (Thermo Scientific, Warsaw, Poland, Cat. No. 12574018), 0.25 µg/mL of bovine serum albumin (BSA), a 1:30,000 dilution of SYBR Green I nucleic acid gel stain (Thermo Scientific, Cat. No. S-7567), and 0.5 µM of PCSK9-specific primers (forward primer: 5′-AGGGGAGGACATCATTGGTG-3′; reverse primer: 5′-CAGGTTGGGGGTCAGTACC-3′ [34]. A reaction mixture from the T7 RNA transcription assay served as a template for RT–qPCR; two microliters of a 100-fold dilution was used.

The thermal profile consisted of the following steps: initial denaturation for 2 min at 95 °C, followed by 45 cycles of 15 s at 95 °C for denaturation and 60 s at 59 °C for annealing.

The amplification steps were followed by the generation of a melting curve that verified the reaction specificity. Each sample was analyzed in triplicate.

Simultaneously, the same approach was achieved by means of conventional PCR followed by gel electrophoresis. In this case, the same reaction components and thermal conditions were used as those previously described for RT–qPCR. Importantly, the number of cycles was reduced to 15 in order to finish the reaction at its logarithmic phase of product amplification. Then, the products were subjected to electrophoresis in an 1.5% agarose gel conducted at a voltage of 80 V for one hour. Following separation, the samples were examined under UV light on the gel visualization station and photographed (BiometraTI1 Transilluminator, Analytik Jena GmbH, Jena, Germany; Cat. No. 057-000). Reverse transcription–real-time PCR (RT–real-time PCR) was performed on a LightCycler 480II sequence analyzer (Roche Diagnostics, Mannheim, Germany), while for conventional PCR, the Mastercycler ep PCR thermocycler (Eppendorf AG, Hamburg, Germany) was used.

### 4.6. In Vitro Synthesis of GFP-Tagged PCSK9 Protein Using Cell Free Transcription/Translation System

Green fluorescent protein (GFP)-tagged PCSK9 was synthesized using cell-free in vitro transcription/translation systems. Two systems, the TNT Quick Coupled Transcription/Translation System and the *E. coli* T7 S30 Extract System for Circular DNA (both from Promega Inc., Wisconsin, MA, USA), were compared to find the best performance for our needs. A circular pCMV6-AC-GFP plasmid containing the coding sequence for PCSK9 (OriGene Technologies, Rockville, MD, USA, Cat No. RG220000) served as a template. The *E. coli* T7 S30 Extract System was found to be more efficient in PCSK9 protein synthesis than the plasmid mentioned above. Therefore, this system was used in further analyses. The composition of the mixture and reaction conditions were those recommended by the manufacturer. Concentrations of particular components in the samples are given in Table 4.

In order to determine the inhibitory efficacy of specific PNA oligomers on the PCSK9 protein synthesis, an amount of 3.0 µM of each PNA was added to the reaction mixture. A PNA oligomer with no complementarity to the *PCSK9* gene was used for control purposes. The experimental design was similar to that of the “In vitro transcription reaction using T7 RNA Polymerase” chapter described above, but only one concentration of 3.0 µM of each PNA was used (Table 4).

### 4.7. Western Blot Analysis of Synthesized GFP-Tagged PCSK9 Protein

After the transcription/translation reaction was finished, protein products were purified by means of centrifugation using a Microcon-30 Centrifugal Filter (Merck Millipore, Poznań, Poland). Residual eluates were diluted with PBS buffer to a final volume of 300 µL. Then, 25 µL of purified products was subjected to denaturation at 96 °C for 6 min, followed by electrophoresis in 10% polyacrylamide gel electrophoresis (PAA). The NEB color protein standard broad range size marker was included in the gel for comparative analysis (New England BioLabs Ipswich, MA, USA, cat. No. P7712). After electrophoretic separation, proteins were electro-transferred onto a Immobilon-P PVDF membrane at 100 mA overnight (Merck Millipore, Poznań, Poland, cat. No. IPVH00010). On the next day, unspecific antibody binding sites were blocked by incubating the membrane in a 3% solution of bovine serum albumin (BSA) for one hour. Subsequently, membranes were placed in a solution containing 3% BSA/1× TTBS (TBS supplemented with 0.05% of Tween-20) and appropriate specific antibodies at a final dilution of 1:1000.

Immunodetection was carried out according to the standard protocol using primary rabbit anti-GFP antibodies (Thermo Scientific, Warsaw, Poland; cat. No. TA150070). Antibody incubation was carried out for one hour at room temperature with continuous rocking. After two washes (10 min each) in 1× TTBS, an anti-rabbit IgG (whole molecule)-peroxidase conjugated secondary antibody (Merck Sigma Aldrich, Poznań, Poland, cat. No. A0545) was added at a dilution of 1:10,000 in 3% BSA/TTBS. This incubation was also performed for one hour under continuous rocking.

Finally, after three washes (two in TTBS for 5 min each and one in TBS for 5 min), a specific chemiluminescent signal was developed using the Pierce ECL Western Blotting Substrate (Thermo Scientific, Warsaw, Poland, cat. No. 32209). Finally, membranes were digitized using the ChemiDoc-It Imaging System (Analytik Jena, Jena, Germany). The integrated optical density measurements representing the protein amount of interest in a sample were performed using ImageJ 1.53t software [25].

### 4.8. High-Performance Liquid Chromatography (HPLC)

The HPLC analysis of the PCSK9 protein after its in vitro synthesis was possible due to the fluorescence emitted by the green fluorescent protein (GFP) that was added at the C end of the PCSK9 chain (GFP-tagged PCSK9). A relative amount of PCSK9 protein concentration in the samples containing different PNA oligomers, as well as in control samples, was analyzed by means of reverse-phase HPLC with fluorescent detection. The Merck/Hitachi D-7000 HPLC System with Hitachi/Merck LaChrom L 7485 Fluorescence Detector was utilized for sample separation together with a Phenomenex Kinetex C18 RP column (d = 4.6 mm, l = 150 mm, particle diameter 2.6 µm) (Shim-Pol, Warsaw, Poland; cat. No. 00F-4462-E0). Analytes were eluted from the column with a mixture of 0.1% trifluoroacetic acid (TFA) in deionized water (Solution A) and 0.1 % TFA in acetonitrile (Solution B). TFA was purchased from Merck Millipore (Poznań, Poland; cat. No. 302031). Detailed HPLC conditions are shown in Table 5.

### 4.9. Statistical Analysis

Data were collated in a Microsoft Excel spreadsheet (v. 1808, Microsoft Corp., Redmond, WA, USA) and transferred to the Statistica software package v. 13.0 (StatSoft Inc., Tulsa, OK, USA) and Plus Set v. 5.0 (TIBCO Software Inc., Palo Alto, CA, USA). The normality of distribution of the variables was evaluated using the Shapiro–Wilk test. Data were compared using ANOVA with Tukey’s HSD post hoc test. Data are expressed as means ± SEM.

## Figures and Tables

**Table 1:** Sequences of synthesized DNA fragments that were used in capillary electrophorese (CE). This sequences encompasses PNA target sites. Detailed information regarding DNA templates is given.

DNA Oligo Name	DNA Sequence (5′→3′)	Length [bp]	GC Content [%]	Melting Temperature [°C]	Orientation
InitDNA_F	AATCAGATAGGATCGTCCGATGGGGCTCTGG	31	54.84	76.02	sense
InitDNA_R	CCAGAGCCCCATCGGACGATCCTATCTGATT	31			antisense
Ex1DNA_F	TGGTGCTGAAGGAGGAGACCCACCTC	26	61.54	76.46	sense
Ex1DNA_R	GAGGTGGGTCTCCTCCTTCAGCACCA	26			antisense
Ex2DNA_F	ACATCGAGGAGGACTCCTCTGTCTTTGCCCA	31	54.84	78.21	sense
Ex2DNA_R	TGGGCAAAGACAGAGGAGTCCTCCTCGATGT	31			antisense

**Table 2:** Sequences of synthesized PNA-Ahx-NLS conjugates (Ahx-6-aminohexanoic acid).

Compound	Sequence
PCSK9-INIT-Ahx-NLS (InitPNA)	G-G-A-T-C-G-T-C-C-G-A-T-G-G-G-Ahx-Pro-Lys-Lys-Lys-Arg-Lys-Val-amide
PCSK9-PPT-EX1-Ahx-NLS (Ex1PNA)	A-G-A-G-G-A-G-G-A-A-G-Ahx-Pro-Lys-Lys-Lys-Arg-Lys-Val-amide
PCSK9-EX2-Ahx-NLS (Ex2PNA)	G-A-G-G-A-G-G-A-C-T-C-C-T-C-T-Ahx-Pro-Lys-Lys-Lys-Arg-Lys-Val-amide
ACLY-INIT-6-Ahx-NLS (CtrlPNA)	A-C-C-G-G-C-T-G-C-T-G-G-G-G-T-Ahx-Pro-Lys-Lys-Lys-Arg-Lys-Val-amide

**Table 3:** Properties of PNA oligomers used in this study.

		Site of Complementarity on the PCSK9 Reference Sequence AY829011.1	
Name	Molecular Mass	Target Localization	Remarks
InitPNA	5137.25	1929–1938	Encompasses start codon
Ex1PNA	4119.32	6337–6347	Within exon 1, polypurine tract
Ex2PNA	5081.22	9010–9024	Within exon 2
CtrlPNA	5113.22	no target within *PCSK9* gene	PNA for control purposes

**Table 4:** Reaction components for cell-free in vitro transcription/translation system.

Reaction Component	Final Amount Per Reaction
DNA template (0.5 ng/µL)	10 µL
Amino Acid Mixture Minus Methionine	2.5 µL
Amino Acid Mixture Minus Cysteine	2.5 µL
T7 S30 Extract, Circular	15 µL
S30 Premix Without Amino Acids	20 µL
TOTAL VOLUME:	50 µL

**Table 5:** High-performance liquid chromatography (HPLC) conditions for analysis of GFP-tagged PCSK9 protein after cell-free synthesis in vitro. The reaction was carried out under the following conditions: flow: 0.8 mL/min; detection (ex/em): 482/511 nm; column temperature: 40 °C; injection volume: 10 µL; mobile phase A: 0.1% trifluoroacetic acid in deionized water; mobile phase B: 0.1% trifluoroacetic acid in acetonitrile.

Gradient	Time (min)	% A	% B
	0.00	65	35
	4.00	55	45
	4.25	55	45
	4.30	65	35
	6.00	65	35

## Data Availability

All relevant data are included in the manuscript.
